# *Myo-*inositol affects the health, metabolome, and lactation performance of peripartum dairy cows

**DOI:** 10.3389/fimmu.2025.1605244

**Published:** 2025-06-16

**Authors:** Chun-zheng Wang, Jia-nan Dong, Wu-wen Sun, Ke Qu, Zi-chen Lin, Natnael D. Aschalew, Yang Zhao, Zhe Sun, Na Ta, Zhi-kun Zhao, Yong-jun Liu, Wei-gang Zhang, Wei Zhao, Gui-xin Qin, Yu-guo Zhen, Tao Wang

**Affiliations:** ^1^ College of Animal Science and Technology, Jilin Agricultural University-Borui Dairy Science and Technology R&D Center, Key Laboratory of Animal Nutrition and Feed Science of Jilin Province, Key Laboratory of Animal Production Product Quality and Security Ministry of Education, Jilin Agricultural University, Changchun, China; ^2^ College of Animal Science and Technology, Ningxia University, Yinchuan, China; ^3^ Postdoctoral Scientific Research Workstation, Feed Engineering Technology Research Center of Jilin Province, Changchun Borui Science & Technology Co., Ltd., Changchun, China; ^4^ College of Agriculture and Natural Resources, Dilla University, Dilla, Ethiopia; ^5^ College of Life Science, Jilin Agricultural University, Changchun, China; ^6^ Tongliao Agricultural and Animal Husbandry Science Research Institute, Tongliao, China; ^7^ Ningxia Agricultural Reclamation Helan Mountain Dairy Co., Ltd., Yinchuan, China

**Keywords:** *myo*-inositol, peripartum dairy cows, metabolomes, milk production, health

## Abstract

**Introduction:**

Yeast culture (YC) has become a crucial microecological preparation for regulating the ruminal environment and improving ruminant health. *Myo*-inositol is an effective substance in YC, our study aimed to investigate whether *myo*-inositol can regulate metabolism and influence the health of dairy cows. Therefore, this study explored the effects of *myo*-inositol on the health, metabolome, and lactation performance of peripartum dairy cows using both *in vitro* and *in vivo* models.

**Methods:**

The ANKOM RFS gas production system was used for the *in vitro* experiment; Twenty four healthy Holstein cows were assigned to CON group (n = 4; no supplementation of YC or *myo*-inositol), YC group (n = 10; 2 g/kg YC), and IN65 group (n = 10; 2 g/kg YC + 16 mg/kg *myo*-inositol) for *in vivo* experiment.

**Results:**

The concentrations of five volatile fatty acids (acetic acid, butyric acid, isobutyric acid, valeric acid, and isovaleric acid), and total volatile fatty acids were significantly higher in the IN65 group compared to the CON group at 6 h *in vitro* (*P* < 0.01). Therefore, 2 g/kg YC plus 16 mg/kg *myo*-inositol was selected for *in vivo* experiment. It was found that the average milk production of the IN65 group increased by 4.00% and 4.05% respectively, compared to the control group and YC group on day 21 postpartum. The levels of superoxide dismutase (SOD), total antioxidant status (TAS), and total oxidative status (TOS) were significantly higher on day 21 compared to day 1 postpartum (*P* < 0.01). However, a trend indicating a decrease in immune function was observed in the IN65 group compared to the CON group. These findings suggested that *myo*-inositol may improve the health of postpartum dairy cows. Additionally, Mantel analysis showed acetic acid was positively correlated with D-glyceric acid and xanthosine. Similarly, D-glyceric acid, inosine 5-monophosphate, and xanthosine were positively correlated with microbiota at the genus, such as *Bulleidia* and *Moryella*.

**Discussion:**

In summary, *myo*-inositol influences energy metabolism by modulating rumen microbial abundance, improving dairy cow health, and increasing milk production.

## Graphical Abstract

Created using Figdraw.

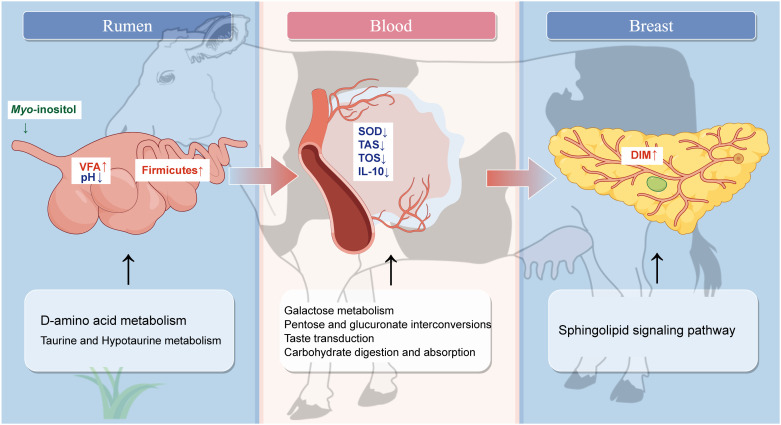

## 1 Introduction

The peripartum period of dairy cows lasts from three weeks before calving to three weeks after calving. This period includes pregnancy, calving, and lactation (1). During this period, the nutritional demands of dairy cows often exceed available nutrients, leading to a negative energy balance (2) and peripartum metabolic disorders (3). This condition is characterized by decreased glucose and insulin levels (4), increased growth hormone concentration (5), excessive mobilization of fat, proteins, glycogen, and minerals; alterations in immune function; and reduced feed intake. During this period dairy cows are immunosuppressive state, making them highly susceptible to diseases such as milk fever, retained placenta, metritis, ketosis, abomasum displacement, lameness, and clinical mastitis (6). Changes in immune cell populations and cytokines, along with reduced antibody levels, contribute to this susceptibility. Suppressive T cells are transported preferentially during calving (7), and postpartum cows rely on T cells for disease resistance (8).

Yeast culture (YC) is a product derived from active yeast through fermentation, concentration, and drying processes. Active yeasts are considered to have probiotic effects, while YC components are believed to have both probiotic and prebiotic effects (9). YC is composed of yeast cell walls (β-glucans and mannan oligosaccharides), soluble cellular substances, vitamins, proteins, peptides, AA, nucleotides, lipids, organic acids, oligosaccharides, esters, and alcohols (10). It can help maintain a healthy rumen environment by regulating pH and reducing the risk of ruminal acidosis (11). Additionally, YC can help balance rumen microbiota (12) and significantly improve the ruminal environment. Over a century and a half ago, German chemist Joseph Scherer first isolated *myo*-inositol from muscle tissue and named it after the Greek word for muscle (13). *Myo*-inositol consists of nine isomers, with *myo*-inositol being the most commonly found isomer (14). Different inositol compounds have significant biochemical importance. The enzymes IRE1α and JNK, which rely on inositol, are involved in metabolic regulation. Targeting the IRE1α-JNK axis can help regulate lipogenesis and fatty acid oxidation, leading to a decrease in lipid accumulation induced by non-esterified fatty acids in bovine hepatocytes (15). As a result, inositol and its components affect fat metabolism in dairy cows. Although there have been many studies on *myo*-inositol in fish, there is a lack of research on ruminants, specifically peripartum dairy cows (16).

The effects of *myo*-inositol on the health and productivity of dairy cows during the peripartum period remain unknown. This study aimed to investigate the effects of *myo*-inositol supplementation on the health, metabolome, and lactation performance of peripartum dairy cows. The hypothesis was that *myo*-inositol would improve milk production and health of peripartum dairy cows.

## 2 Materials and methods

### 2.1 Experimental design and animal husbandry

Animal experiments, both *in vitro* and *in vivo*, were conducted in accordance with the guidelines approved by the Jilin Agricultural University Animal Research Ethics Committee (approval number: JLAU-ACUC2022-004).

#### 2.1.1 *In vitro* experiment

Ruminal fluid was collected from three dairy cows with ruminal fistulas, all of which had free access to clean water and fed a TMR. The *in vitro* experiment was followed a single-factor design and included six treatment groups: CON, YC, IN65, IN85, IN125, and IN205. The CON group did not supplement with any YC or *myo-*inositol. The YC group was supplemented with 2 g/kg YC (per kilogram of feed), the IN65 group with 2 g/kg YC (per kilogram of feed) + 16 mg/kg *myo-*inositol (per kilogram of feed), the IN85 group with 2 g/kg YC (per kilogram of feed) + 36 mg/kg *myo-*inositol (per kilogram of feed), the IN125 group with 2 g/kg YC (per kilogram of feed)+ 76 mg/kg *myo-*inositol (per kilogram of feed), and the IN205 group with 2 g/kg YC (per kilogram of feed) + 156 mg/kg *myo-*inositol (per kilogram of feed). Our previous studies found that the *myo*-inositol content was 49 mg in 2 g YC (17). Therefore, the *myo-*inositol levels in the CON, YC, IN65, IN85, IN125, and IN205 groups were 0, 49, 65, 85, 125, and 205 mg/kg, respectively. Details of the basal diets are presented in **Table 1**.

**Table 1:** Ingredients and nutrient composition of the basal diet.

Ingredients	% DM^1^	Nutrient levels^3^	% DM
Alfalfa hay	2.39	CP	16.00
Oat hay	5.83	EE	5.10
Corn	17.00	NDF	30.16
Flaked corn	7.67	ADF	17.63
Corn silage	35.11	Starch	30.00
Cotton seed	2.83	P	0.38
Cotton seed meal	6.44	Ca	0.80
Soybean meal	7.00	NE_L_^4^ (mCal/kg)	1.78
Beet granules	3.11	RUP^5^ (%CP)	36.99
Fat	1.72	RDP (%CP)	63.01
NaHCO_3_	0.89		
Molasses	3.39		
5%Lactation premix^2^	6.67		
Total	100		

^1^DM, dry matter.

^2^Provided per kilogram of total mixed ration (on DM basis): VA 516 KIU, VD 106 KIU, VE 6814 IU, nicotinamide 6815 IU, Cu 592 mg, Zn 2353 mg, Mn 1650 mg, Co 16.1 mg, I 25.7 mg, Se 34.0 mg.

^3^Measured values.

^4^Estimated based on NRC (2001). NEL, Net energy required for lactation.

^5^RUP and RDP calculated with CPM-Dairy.

The *myo*-inositol (purity > 99%, CAS: 87-89-8) used in the *in vitro* experiments was purchased from Anhui Yuanzheng Biological (Anhui, China). Before morning feeding, rumen fluid was collected through ruminal cannulas from dairy cows, ensuring sampling from multiple rumen locations. Immediately after collection, rumen fluid was transferred into thermos bottles preheated to 39°C and pre-flushed with CO_2_ to maintain anaerobic conditions. In the laboratory, rumen fluid was filtered through four-layer cheesecloth and mixed with buffer solution at a ratio of 1:2, and then divided into culture bottles and incubated in a 40 °C water bath shaker (80 rpm), utilized 120 bottles [one substrates × six doses (0, 49, 65, 85, 125, and 205 mg/kg) × four time points (3, 6, 12, or 24 h) × five replicates]. Detailed experimental procedures and buffer solution preparation refer to Dong et al. (18).

#### 2.1.2 *In vivo* experiment

In this study, 24 healthy Holstein cows in the early stages of pregnancy were carefully selected from a commercial dairy farm in Ning Xia, China, based on similar body condition scores (3.0 – 3.5) and met the necessary quarantine requirements. The feeding management and dietary composition were consistent with those used in the *in vitro* experiments. Twenty-four cows were randomly assigned to 3 groups as follows: CON (n = 4; no supplementation of YC or *myo*-inositol), YC (n = 10; 2 g/kg YC), and IN65 (n = 10; 2 g/kg YC + 16 mg/kg *myo-*inositol). The YC and *myo*-inositol were supplemented and mixed uniformly in the TMR. They were housed in large groups, and the barn was cleaned three times daily to provide ample drinking water, sodium bicarbonate, and salt. The cowshed is sufficiently spacious to allow the cows to move freely, and the temperature within the cowshed is maintained at 10°C ± 2°C.

### 2.2 Collection of rumen fluid samples for *in vitro* experiments

Rumen fermentation fluid samples were collected after 3, 6, 12, and 24 h of cultivation and divided into five parts: 1.5 mL for VFA concentration, 1 mL for ammonia nitrogen (NH_3_-N) concentration, 2 mL for microbial flora analysis, and 2 mL for metabolomics. The remaining fluid was used for pH determination. Samples for VFA and NH_3_-N determination were frozen at -40°C, while those for microbial flora and metabolomics were frozen at -80°C. Rumen fluid for VFA determination was centrifuged at 10,000 g for 10 minutes at 4°C. Subsequently, 1 mL of the supernatant and 0.2 mL of a 25% metaphosphate solution were added to a 2 mL centrifuge tube and allowed to precipitate overnight. Afterward, the mixture was centrifuged again at 10,000 g for 10 minutes at 4°C, and the supernatant was collected in a new 2 mL centrifuge tube for further analysis.

### 2.3 Collection of milk and serum samples for *in vivo* experiments

#### 2.3.1 Milk

The cows were milked using an automated system, and milk production was recorded manually. Approximately 10 mL of milk was collected each time and stored in a liquid nitrogen tank. Milk samples were collected on the 1^st^, 7^th^, 14^th^, and 21^st^ days after delivery. Before pretreatment, 1 mL of milk sample was collected at three time points (morning, mid-day, and evening) and mixed for metabolomic analysis.

#### 2.3.2 Serum

Blood samples were collected from the tail vein on the day of calving and 21 days postpartum. The samples were processed in vacutainers to measure inflammation, immunity, antioxidant levels, and metabolome. After centrifugation at 3000 ×g for 15 min at 4°C, the samples were stored in liquid nitrogen for further analysis. Processing was completed within 30 min.

### 2.4 Determination of rumen fermentation parameters *in vitro* and serum

VFA concentrations were measured using a gas chromatograph (7890 B; Agilent Technologies, Santa Clara, CA, USA) (19). The pH was measured using a pH meter (MP523-04; Shanghai Sanxin Instrument Co. Ltd., Shanghai, China). NH_3_-N levels were quantified using a spectrophotometer (UV-1201, Shimadzu Corporation, Kyoto, Japan) (20).

Blood indicators including inflammatory ( IL-6, IL-10, IL-1β, TNF-α, and IFN-γ), immune (IgA and IgM), and oxidation/antioxidant (CAT, MDA, GSH-Px, SOD, TAS, and TOS). Indicators were measured using enzyme-linked immunosorbent assay kits (Lengton, Shanghai, China), and the tests were performed in accordance with the manufacturer’s instructions.

### 2.5 Determination of rumen microbial community *in vitro*

The bacterial 16S rRNA gene V3–V4 region was amplified by PCR using the forward primer (F): ACTCCTACGGGAGGCAGCA and reverse primer (R): GGACTACHVGGGTWTCTAAT. Sample-specific 7-bp barcodes were included in the primers for multiplex sequencing. The PCR mixture included 5 μL of buffer (5×), 0.25 μL of fast pfu DNA polymerase (5U/μL), 2 μL (2.5 mM) of dNTPs, 1 μL (10 µM) of each forward and reverse primer, 1 μL of DNA template, and 14.75 μL of ddH_2_O. The thermal cycling protocol consisted of an initial denaturation at 98°C for 5 min, followed by 25 cycles of denaturation at 98°C for 30 s, annealing at 53°C for 30 s, and extension at 72°C for 45 s, with a final extension at 72°C for 5 min. PCR amplicons were purified using Vazyme VAHTSTM DNA Clean Beads (Vazyme, Nanjing, China), quantified with a Quant-iT PicoGreen dsDNA Assay Kit (Invitrogen, Carlsbad, CA, USA), and then pooled in equal amounts for pair-end 250-bp sequencing on the Illumina NovaSeq platform with the NovaSeq 6000 SP Reagent Kit (500 cycles) at Shanghai Personal Biotechnology Co., Ltd (Shanghai, China). Tax4Fun was employed to predict functional characteristics consolidated according to the Kyoto Encyclopedia of Genes and Genomes (KEGG) pathway classification system. The data presented in the study are deposited in the NCBl Sequence Read Archive repository, accession number PRJNA1101182.

### 2.6 Metabolomics analysis methods

The sample (100 μL) was mixed with an extraction solution (MeOH: ACN, 1:1[v/v], 400 μL) containing deuterated internal standards. The mixture was vortexed for 30 s, sonicated for 10 min in a 4°C water bath, and incubated for 1 h at 40°C for 1 h to precipitate proteins. After centrifugation at 12000 rpm (RCF=13800 [× g], R=8.6 cm) for 15 min at 4°C, the supernatant was transferred to a fresh glass vial for further analysis. Quality control samples were prepared by mixing equal aliquots of the supernatants.

LC-MS analyses were performed using an Ultra-High Performance Liquid Chromatography system (Vanquish, Thermo Fisher Scientific) with a Waters ACQUITY UPLC BEH Amide (2.1 mm × 50 mm, 1.7 μm) coupled to Orbitrap Exploris 120 mass spectrometer (Orbitrap MS, Thermo). The mobile phase consisted of 25 mmol/L ammonium acetate and 25 mmol/L ammonia hydroxide in water (pH 9.75) (A) and acetonitrile (B). The auto-sampler temperature was 4°C, and the injection volume was 2 μL. An Orbitrap Exploris 120 mass spectrometer was used to acquire MS/MS spectra in information-dependent acquisition mode under the control of an acquisition software (Xcalibur, Thermo). In this mode, the acquisition software continuously evaluated the full-scan MS spectrum. The Electrospray ionization source conditions were set as follows: sheath gas flow rate at 50 Arb, Aux gas flow rate at 15 Arb, capillary temperature at 320°C, full MS resolution at 60000, MS/MS resolution at 15000, collision energy: SNCE 20/30/40, and spray voltage at 3.8 kV (positive) or 3.4 kV (negative), respectively.

The raw data were converted to the mzXML format using ProteoWizard and subsequently processed with an in-house program developed in R, which is based on XCMS for peak detection, extraction, alignment, and integration.

### 2.7 Statistical analysis

Data were analyzed using SPSS Statistics software (version 22.0, IBM). The experiment used a one-way analysis of variance for data analysis. Spearman’s correlation coefficient was utilized to analyze the correlation between microbial communities at the genus level and fermentation indicators, and Mantel’s test was used to explore the relationship between differential metabolites, microbial communities, and fermentation indicators.

## 3 Results

### 3.1 Analysis of rumen fermentation parameters *in vitro*

The effects of *myo*-inositol on *in vitro* ruminal fermentation are summarized in **Table 2**. After 3 h of *in vitro* fermentation, the pH of the YC, IN65, IN85, and IN205 groups were significantly lower than the CON group (*P* < 0.01). After 6 h of *in vitro* fermentation, the pH of the IN85 and IN205 groups were significantly lower compared to the CON group (*P* < 0.05). After 12 h of *in vitro* fermentation, the pH of the IN125 group was significantly lower than that of the YC group (*P* < 0.01). Following 24 h of *in vitro* fermentation, the pH of the YC, IN65, and IN85 groups were significantly higher than that of the CON group (*P* < 0.01).

**Table 2:** Effects of YC and *myo*-inositol supplementation on pH, NH_3_-N (mg/100 mL) and VFAs concentration (mmol/L) *in vitro*.

Item	Treatments						SEM	*P*-Value
	CON	YC	IN65	IN85	IN125	IN205		
pH								
0 h	7.14	7.14	7.14	7.14	7.14	7.14	Null	Null
3 h	7.12^Aa^	6.92^BCb^	6.89^Cb^	6.93^BCb^	7.04^ABa^	6.88^Cb^	0.02	<0.01
6 h	6.94^a^	6.86^ab^	6.88^a^	6.84^b^	6.95^a^	6.83^b^	0.01	0.03
12 h	6.79^ABabc^	6.81^Aa^	6.79^ABab^	6.80^Aab^	6.77^Bc^	6.78^ABbc^	0.00	<0.05
24 h	6.64^Cc^	6.71^Aa^	6.69^ABab^	6.68^ABab^	6.66^BCbc^	6.67^Cac^	0.01	<0.01
NH_3_-N mg/100 mL								
0 h	7.52	7.52	7.52	7.52	7.52	7.52	Null	Null
3 h	9.84^Cc^	11.57^Bb^	11.31^Bb^	10.95^Bb^	13.62^Aa^	13.01^Aa^	0.28	<0.01
6 h	14.03^Bb^	14.21^Bb^	14.64^Bb^	16.75^Aa^	16.90^Aa^	13.78^Bb^	0.30	<0.01
12 h	12.92^Bb^	16.32^Aa^	12.14^Bb^	14.06^ABb^	13.30^Bb^	13.83^ABb^	0.36	<0.01
24 h	22.69	24.13	21.83	22.52	23.80	23.40	0.29	0.31
Acetic acid mmol/L								
0 h	6.57	6.57	6.57	6.57	6.57	6.57	Null	Null
3 h	22.97	19.33	22.85	21.04	28.46	25.02	0.97	0.09
6 h	30.77^Bb^	30.44^Bb^	52.61^Aa^	39.38^Bb^	29.61^Bb^	33.48^Bb^	2.02	<0.01
12 h	49.18^Bb^	43.85^Bbc^	60.82^Aa^	60.79^Aa^	46.28^Bbc^	42.06^Bc^	1.74	<0.01
24 h	51.67	63.67	61.22	60.85	55.86	52.50	1.87	0.32
Propionic acid mmol/L								
0 h	4.15	4.15	4.15	4.15	4.15	4.15	Null	Null
3 h	4.95^b^	4.82^b^	6.65^a^	5.39^ab^	6.73^a^	6.46^a^	0.23	0.02
6 h	6.89	7.57	8.25	10.64	7.98	8.18	0.37	0.06
12 h	12.58^BCb^	12.62^BCb^	18.16^Aa^	16.60^ABa^	12.43^BCb^	11.96^Cb^	0.62	<0.01
24 h	27.53^Cb^	26.10^Cb^	37.11^ABa^	36.97^ABa^	42.18^Aa^	30.15^BCb^	1.40	<0.01
Butyrate acid mmol/L								
0 h	3.11	3.11	3.11	3.11	3.11	3.11	Null	Null
3 h	3.41^BCb^	3.31^Cb^	4.76^ABa^	4.13^ABCab^	4.72^ABa^	4.91^Aa^	0.18	<0.01
6 h	5.13^Cc^	6.64^BCc^	13.06^Aa^	8.68^Bb^	6.45^BCc^	6.51^BCc^	0.59	<0.01
12 h	10.00^BCb^	9.01^Cb^	14.10^Aa^	12.69^ABa^	9.97^BCb^	9.71^BCb^	0.47	<0.01
24 h	14.37^ABbc^	13.03^Bc^	16.30^ABab^	17.77^Aa^	12.91^Bc^	13.25^Bc^	0.50	<0.01
Isobutyric acid mmol/L								
0 h	0.48	0.48	0.48	0.48	0.48	0.48	Null	Null
3 h	0.58^ab^	0.52^b^	0.73^a^	0.59^ab^	0.69^a^	0.72^a^	0.02	0.03
6 h	0.78^Bc^	1.04^ABbc^	1.41^Aa^	1.19^ABab^	0.99^ABbc^	1.20^ABab^	0.05	<0.01
12 h	1.40^Bb^	1.31^Bb^	1.81^Aa^	1.77^Aa^	1.41^Bb^	1.33^Bb^	0.05	<0.01
24 h	1.95	2.17	2.10	2.25	2.46	2.31	0.07	0.21
Valeric acid mmol/L								
0 h	0.31	0.31	0.31	0.31	0.31	0.31	Null	Null
3 h	0.42^Bbc^	0.39^Bc^	0.52^ABbc^	0.57^ABab^	0.51^ABbc^	0.67^Aa^	0.03	<0.01
6 h	0.66^Cc^	0.84^Cc^	1.76^Aa^	1.28^Bb^	0.88^Cc^	0.80^Cc^	0.08	<0.01
12 h	1.41^BCb^	1.28^Cb^	2.03^Aa^	1.89^ABa^	1.37^BCb^	1.35^BCb^	0.08	<0.01
24 h	1.79	2.16	2.30	2.36	2.16	2.06	0.10	0.79
Isovaleric acid mmol/L								
0 h	0.87	0.87	0.87	0.87	0.87	0.87	Null	Null
3 h	0.93	1.00	1.05	1.04	1.03	1.26	0.04	0.22
6 h	1.38^Cc^	1.74^BCbc^	3.10^Aa^	2.10^Bb^	1.58^BCc^	1.75^BCbc^	0.13	<0.01
12 h	2.50^BCb^	2.21^Cb^	3.45^Aa^	3.15^Aab^	2.43^BCb^	2.40^BCb^	0.11	<0.01
24 h	3.69	4.11	4.23	3.43	4.26	4.07	0.12	0.33
Total volatile fatty acids (6 types) mmol/L								
0 h	14.69	14.69	14.69	14.69	14.69	14.69	Null	Null
3 h	30.40	29.22	39.99	32.76	39.68	40.29	1.50	0.05
6 h	45.62^Bc^	47.06^Bc^	87.07^Aa^	63.27^Bb^	47.43^Bc^	51.79^Bbc^	3.51	<0.01
12 h	73.65^ABCb^	70.27^BCb^	87.32^ABa^	89.42^Aa^	72.01^ACb^	68.82^Cb^	2.26	<0.01
24 h	84.11	101.33	91.06	105.37	93.49	88.34	2.76	0.21

Different superscript uppercase letters within a row indicate significant differences between treatment groups (*P* < 0.01). Different superscript lowercase letters within a row indicate significant differences between treatment groups (*P* < 0.05). And if the superscript letters is same or no letter mark indicates that the difference is not significant (*P* > 0.05), the following **Tables 3**–**6** is the same.

**Figure 1:**
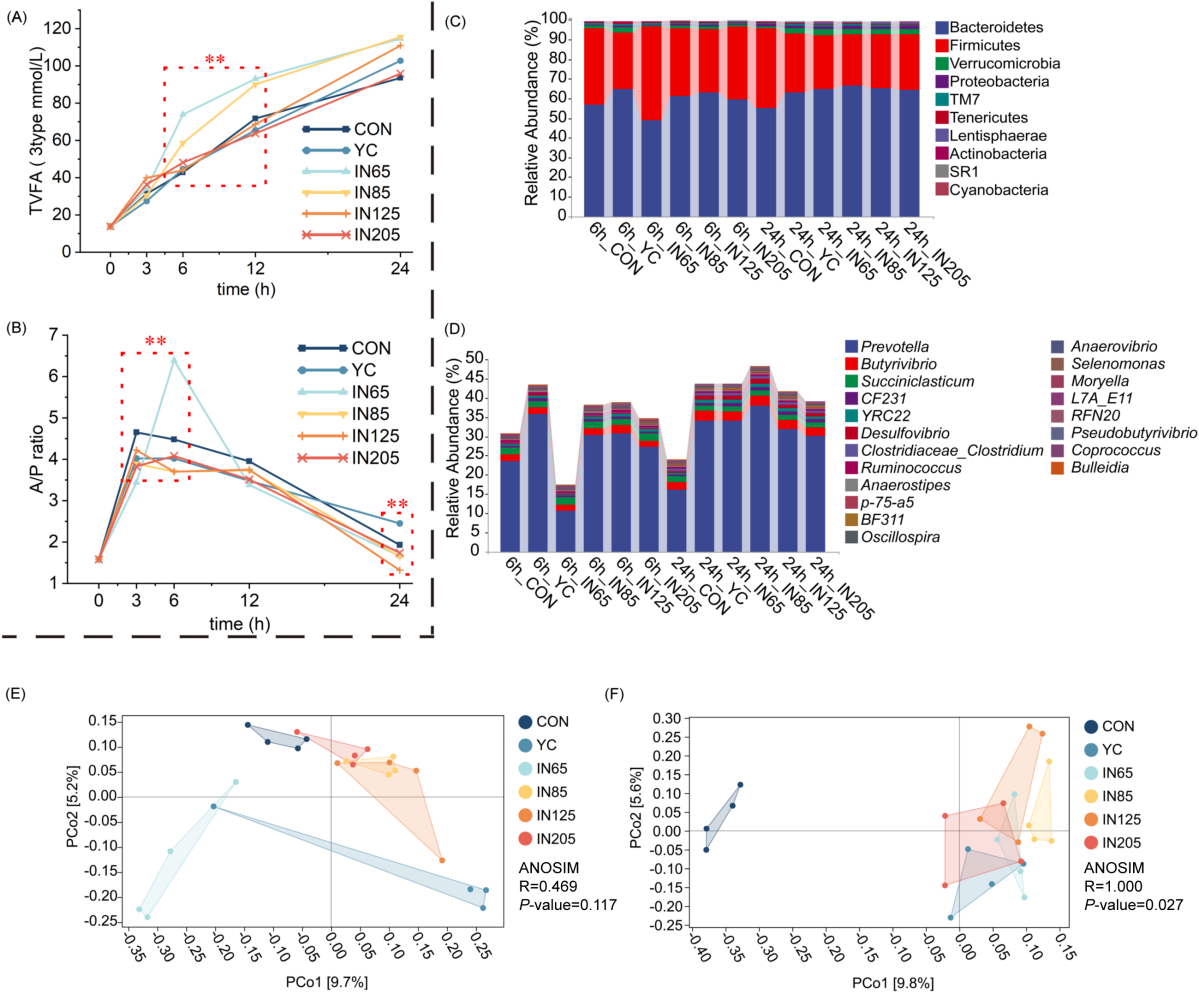
Effects of YC and *myo*-inositol supplementation on TVFA (3 types) **(A)**, A/P ratio **(B)**, ruminal bacterial community at phylum levels **(C)** and genus levels **(D)**, the beta diversity at 6 h **(E)** and 24 h **(F)** *in vitro*.

After 3 h of *in vitro* fermentation, NH_3_-N concentrations in YC, IN65, IN85, IN125, and IN205 were significantly higher than those in the CON group (*P* < 0.01), with NH_3_-N concentrations in IN125 and IN205 being significantly higher than those in the YC group (*P* < 0.01). After 6 h of *in vitro* fermentation, NH_3_-N concentrations in IN85 and IN125 were significantly higher than those in the CON and YC groups (*P* < 0.01). After 12 h of *in vitro* fermentation, the NH_3_-N concentration in the YC group was significantly higher than that in the CON group (*P* < 0.01). Notably, after 6 h of in vitro fermentation, the acetic acid/propionic acid ratio (A/P ratio), six VFA, 3type TVFA and 6type TVFA in the IN65 group were significantly higher than those in the CON group (**Table 2**; **Figures 1A, B**).

### 3.2 Analysis of rumen microbial composition *in vitro*

Alpha diversity analysis was conducted to assess the effect of YC and *myo-*inositol on rumen microbial abundance. The Chao1, Faith_pd, Shannon, and Simpson indices were used to evaluate the richness, diversity, and evenness of the microbiota. After 6 h of *in vitro* fermentation, the Shannon index was significantly higher in the IN65 group compared to the YC group (*P* < 0.05). After 24 h of *in vitro* fermentation, the Chao1 and Faith_pd indices were significantly lower in the IN85 and IN125 groups compared to the YC group (*P* < 0.01). The Simpson indices of the YC, IN65, IN85, IN125, and IN205 groups were all significantly higher than the CON group, with the IN85 group showing a significantly lower Simpson index than the YC group (*P* < 0.01; **Table 3**).

**Table 3:** Effects of YC and *myo*-inositol supplementation on alpha diversity of rumen bacterial communities *in vitro*.

Item	Treatments						SEM	P-Value
	CON	YC	IN65	IN85	IN125	IN205		
6 h								
Chao1	7328.25	6866.96	8421.61	5809.72	6121.07	7432.66	276.91	0.06
Faith_pd	251.59^AaBb^	223.37^Bb^	288.70^Aa^	217.07^Bb^	219.89^Bb^	252.25^AaBb^	7.00	<0.01
Shannon	10.39^ab^	10.02^c^	10.46^a^	10.06^bc^	10.00^c^	10.30^abc^	0.06	0.04
Simpson	99.62	99.41	99.47	99.54	99.50	99.60	0.00	0.14
24 h								
Chao1	6416.41^ABa^	6910.86^Aa^	6198.26^ABa^	4736.47^Bb^	4765.66^Bb^	6969.73^Aa^	252.80	<0.01
Faith_pd	257.73^Aa^	223.44^ABb^	207.11^BCb^	174.79^Cc^	175.44^Cc^	224.89^ABb^	6.96	<0.01
Shannon	10.32^Aa^	10.34^Aa^	10.21^ABa^	9.88^Cb^	9.99^BCb^	10.31^Aa^	0.04	<0.01
Simpson	99.57^Cc^	99.72^Aa^	99.73^Aa^	99.64^Bb^	99.70^Aa^	99.73^Aa^	0.01	<0.01

Simpson results were multiplied by 10^3^.

The 16S rRNA gene was sequenced using amplicon sequencing, resulting in 3,473,478 high-quality sequences. On average, each sample had 289,457 sequences. The main microbial phyla consisted of 10 taxa with relative abundances > 0.10%. The most abundant phyla were Bacteroidetes (61.10%), Firmicutes (33.29%), Verrucomicrobia (1.84%), and Proteobacteria (1.11%) (**Figure 1C**). At the genus level, the main microbiota included 20 genera with relative abundances exceeding 0.10%. The most abundant genera were *Prevotella* (28.51%), *Butyrivibrio* (2.10%), and *Succinivibrio* (1.51%) (**figure 1D**).

As shown in **Table 4**, the microbiota at the phylum level were significantly affected by YC and *myo-*inositol. After 6 h of *in vitro* fermentation, the Firmicutes abundance in the IN65 group was significantly higher compared to the YC group (*P* < 0.01), whereas the relative abundances of Bacteroidetes and Lentisphaerae were significantly lower than those in the YC group (*P* < 0.01). After 24 h *in vitro* fermentation, the IN65 group showed significantly higher relative abundances of Bacteroidetes, Verrucomicrobia, Proteobacteria, and Lentisphaerae compared to the CON group (*P* < 0.01), whereas the relative abundances of Firmicutes, and SR1 were significantly lower than that in the CON group (*P* < 0.01). As shown in **Table 5**, at 6 h of *in vitro*, the IN65 group showed a significantly higher relative abundance of *Moryella* at the genus level compared to the YC group and CON group (*P* < 0.01); at 24 h of *in vitro*, the relative abundances of *Prevotella*, *CF231, BF311, YRC22, Selenomonas*, and *Desulfovibrio* in the IN65 group were significantly higher than that in the CON group (*P* < 0.01).

**Table 4:** Effects of YC and *myo*-inositol supplementation on rumen bacterial communities at phylum levels *in vitro*.

Phylum	Treatments						SEM	*P*-Value
	CON	YC	IN65	IN85	IN125	IN205		
6 h								
Bacteroidetes	566.85^ABb^	648.50^Aa^	488.82^Bc^	611.31^Aab^	629.00^Aa^	593.20^Aab^	13.05	< 0.01
Firmicutes	390.21^ABb^	288.60^Bc^	476.45^Aa^	346.93^Bbc^	325.73^Bbc^	370.53^Bb^	14.93	< 0.01
Verrucomicrobia	14.93	29.27	4.29^a^	10.75^b^	11.79^b^	12.12	1.48	< 0.01
Proteobacteria	0.06^Cc^	0.12^Aa^	0.05^Cc^	0.08^BCb^	0.09^ABb^	0.05^Cc^	0.00	< 0.01
TM7	6.28^ABab^	7.46^Aa^	5.76^ABb^	5.44^Bb^	5.79^ABb^	5.66^ABb^	0.21	0.04
Tenericutes	6.42	7.30	6.43	6.13	5.42	5.14	0.27	0.23
Lentisphaerae	0.66^Bbc^	1.66^Aa^	0.18^Bc^	0.83^ABbc^	1.14^ABab^	0.69^ABbc^	0.13	< 0.01
Actinobacteria	2.50	2.38	3.04	2.88	3.50	2.40	0.17	0.36
SR1	1.84	1.60	1.74	2.02	2.27	2.02	0.07	0.09
Cyanobacteria	0.26	0.72	0.09	0.46	0.72	0.27	0.09	0.20
24 h								
Bacteroidetes	552.64^Bb^	628.95^Aa^	649.13^Aa^	667.61^Aa^	653.02^Aa^	643.13^Aa^	9.47	< 0.01
Firmicutes	402.46^Aa^	303.40^Bb^	272.21^Bb^	259.85^Bb^	273.62^Bb^	284.24^Bb^	11.92	< 0.01
Verrucomicrobia	12.41^b^	24.90^a^	29.98^a^	23.87^a^	25.06^a^	27.40^a^	1.73	0.04
Proteobacteria	9.61^Dd^	14.93^BCbc^	16.68^Bb^	20.40^Aa^	15.29^BCbc^	13.86^Cc^	0.71	< 0.01
TM7	4.84^Bb^	5.19^Bb^	6.03^Bb^	6.58^ABb^	8.67^Aa^	6.44^ABb^	0.33	< 0.01
Tenericutes	4.18	4.09	4.20	4.15	5.16	4.12	0.31	0.93
Lentisphaerae	1.96^Bb^	6.91^ABa^	8.83^Aa^	6.48^ABa^	7.07^ABa^	7.45^Aa^	0.62	< 0.05
Actinobacteria	2.58	1.37	1.23	1.08	1.30	1.82	0.13	< 0.05
SR1	1.64^Aa^	1.22^ABCb^	1.00^Cb^	1.18^BCb^	1.65^Aa^	1.58^ABa^	0.07	< 0.01
Cyanobacteria	0.55^Bb^	1.65^Aa^	1.96^Aa^	2.24^Aa^	2.19^Aa^	2.00^Aa^	0.14	< 0.01

**Table 5:** Effects of YC and *myo*-inositol supplementation on ruminal bacterial community at genus levels *in vitro*.

Phylum	Genus	Treatments						SEM	*P*-Value
		CON	YC	IN65	IN85	IN125	IN205		
6 h									
Bacteroidetes	*Prevotella*	236.00^Bb^	358.40^Aa^	106.10^Cc^	302.50^ABab^	307.40^ABab^	272.20^ABab^	19.27	< 0.01
	*CF231*	5.40^BCbc^	8.70^Aa^	3.60^Cc^	8.10^Aa^	7.00^ABab^	5.50^BCbc^	0.43	< 0.01
	*BF311*	0.20^b^	0.40^ab^	0.20^b^	0.50^ab^	0.70^a^	0.30^b^	0.05	0.03
	*YRC22*	4.70^BCb^	6.10^Aa^	3.80^Cc^	6.10^Aa^	5.60^ABa^	6.40^Aa^	0.22	< 0.01
	*Moryella*	1.70^Bb^	0.70^Bc^	3.20^Aa^	1.60^Bb^	1.10^Bbc^	1.60^Bb^	0.19	< 0.01
Firmicutes	*Ruminococcus*	5.30^ab^	3.50^b^	4.40^b^	5.10^ab^	6.80^a^	4.70^b^	0.31	0.03
	*Butyrivibrio*	17.30	16.10	16.80	18.30	23.10	15.20	0.93	0.17
	*p-75-a5*	3.00	2.50	3.00	3.10	2.50	2.70	0.10	0.50
	*Succiniclasticum*	16.60^bc^	15.90^bc^	18.50^ab^	16.40^bc^	14.30^c^	19.60^a^	0.50	0.01
	*Anaerovibrio*	1.20^bc^	2.60^a^	0.70^c^	1.60^ab^	1.70^bc^	2.00^bc^	0.16	0.02
	*Selenomonas*	1.00^Bbc^	2.80^Aa^	0.50^Bc^	2.10^ABab^	2.00^ABab^	2.20^ABab^	0.21	< 0.01
	*Oscillospira*	2.30^a^	1.40^b^	2.30^a^	1.90^ab^	2.00^ab^	2.10^a^	0.10	0.043
	*Clostridiaceae_Clostridium*	1.70^abc^	1.30^bc^	2.10^a^	1.40^bc^	1.20^c^	1.80^ab^	0.09	0.02
	*Anaerostipes*	2.30^bc^	3.50^a^	1.50^c^	2.80^ab^	2.20^bc^	2.30^bc^	0.18	0.02
	*Coprococcus*	1.00	0.70	1.20	1.00	1.60	1.10	0.08	0.05
	*Bulleidia*	1.50^ABb^	0.90^Bc^	2.00^Aa^	1.20^Bbc^	1.20^Bbc^	1.30^Bbc^	0.09	< 0.01
	*Pseudobutyrivibrio*	0.40	0.60	0.30	0.60	0.50	0.30	0.04	0.16
Proteobacteria	*Desulfovibrio*	2.60^Bb^	4.90^Aa^	1.80^Bb^	3.10^Bb^	2.90^Bb^	2.30^Bb^	0.26	< 0.01
Unknown phylum	*L7A_E11*	1.90^a^	1.10^b^	2.20^a^	1.90^a^	1.60^ab^	2.00^a^	0.11	0.04
	*RFN20*	0.90	1.80	0.30	1.50	3.00	0.90	0.29	0.08
24 h									
Bacteroidetes	*Prevotella*	161.40^Cd^	339.80^ABb^	339.50^ABb^	378.70^Aa^	318.60^Bbc^	300.60^Bc^	14.81	< 0.01
	*CF231*	4.40^Cc^	7.40^Bb^	7.70^Bb^	8.30^ABb^	9.70^Aa^	8.40^ABab^	0.37	< 0.01
	*BF311*	1.30^Aa^	3.50^Bb^	3.50^Bb^	3.60^Bb^	3.80^Bb^	4.40^Bb^	0.24	< 0.01
	*YRC22*	4.40^Bc^	8.70^Aab^	8.00^Aab^	8.70^Aa^	7.50^Aab^	7.50^Ab^	0.33	< 0.01
	*Moryella*	1.80^a^	1.40^b^	1.20^b^	1.30^b^	1.40^b^	1.10^b^	0.06	0.01
Firmicutes	*Ruminococcus*	4.10	3.40	3.60	3.70	4.50	4.40	0.18	0.34
	*Butyrivibrio*	19.70	26.90	24.30	27.00	24.40	23.10	0.88	0.14
	*p-75-a5*	2.30^b^	2.00^b^	2.30^b^	2.50^ab^	3.00^a^	2.10^b^	0.10	0.04
	*Succiniclasticum*	14.30	13.10	14.60	13.40	12.90	12.00	0.39	0.46
	*Anaerovibrio*	1.90^Bbc^	3.60^Bc^	3.60^ABb^	3.30^Aa^	4.10^ABb^	3.10^Bbc^	0.17	< 0.01
	*Selenomonas*	1.20^Bc^	1.60^ABab^	1.90^Aa^	1.90^Aa^	1.50^ABabc^	1.50^ABbc^	0.07	< 0.01
	*Oscillospira*	2.70^a^	1.60^b^	1.20^b^	1.10^b^	1.70^b^	1.70^b^	0.15	0.01
	*Clostridiaceae_Clostridium*	7.00	8.10	7.20	8.20	7.60	6.20	0.21	0.06
	*Anaerostipes*	1.60^Bc^	1.50^Aab^	1.90^Aab^	2.20^Aab^	1.90^Aa^	1.70^Ab^	0.06	< 0.01
	*Coprococcus*	1.40^a^	1.00^ab^	0.80^b^	0.80^b^	1.00^ab^	1.10^ab^	0.07	0.03
	*Bulleidia*	0.90	0.80	0.60	0.70	0.70	0.60	0.05	0.72
	*Pseudobutyrivibrio*	1.30	20.00	1.70	2.10	1.80	1.70	0.08	0.06
Proteobacteria	*Desulfovibrio*	5.10^Cd^	9.70^ABbc^	11.00^ABab^	12.60^Aa^	8.40^Bc^	7.90^BCc^	0.57	< 0.01
Unknown phylum	*L7A_E11*	1.80^Aa^	0.80^Bb^	0.60^Bb^	0.60^Bb^	0.70^Bb^	0.80^Bb^	0.11	< 0.01
	*RFN20*	0.60	0.80	0.90	1.20	1.10	1.20	0.08	0.19

Jaccard dissimilarity analysis showed significant differences in microbiota composition among six groups with the same fermentation time. The addition of YC and *myo-*inositol affected the richness, diversity, and uniformity of the rumen microbial communities. At 6 h of *in vitro* fermentation, the YC group closely resembled was close to the IN65 group, while the other four groups clustered together, suggesting that adding a small amount of *myo*-inositol and a specific amount of YC had a significant effect on rumen microorganisms. The IN85 group, IN125 group, IN205 group, and YC group were similar to the CON group, indicating a minimal effect of these four groups on rumen microorganisms. At 24 h of *in vitro*, the treatment groups with *myo*-inositol addition showed similarities but differed significantly from the CON group, suggesting little variation among the IN65 group, the IN85 group, the IN125 group, and the IN205 group, and a large difference between CON and the treatment groups with *myo*-inositol addition (**Figures 1E, F**).

### 3.3 Effects of *myo-*inositol on milk yield and serum parameters

**Figure 2:**
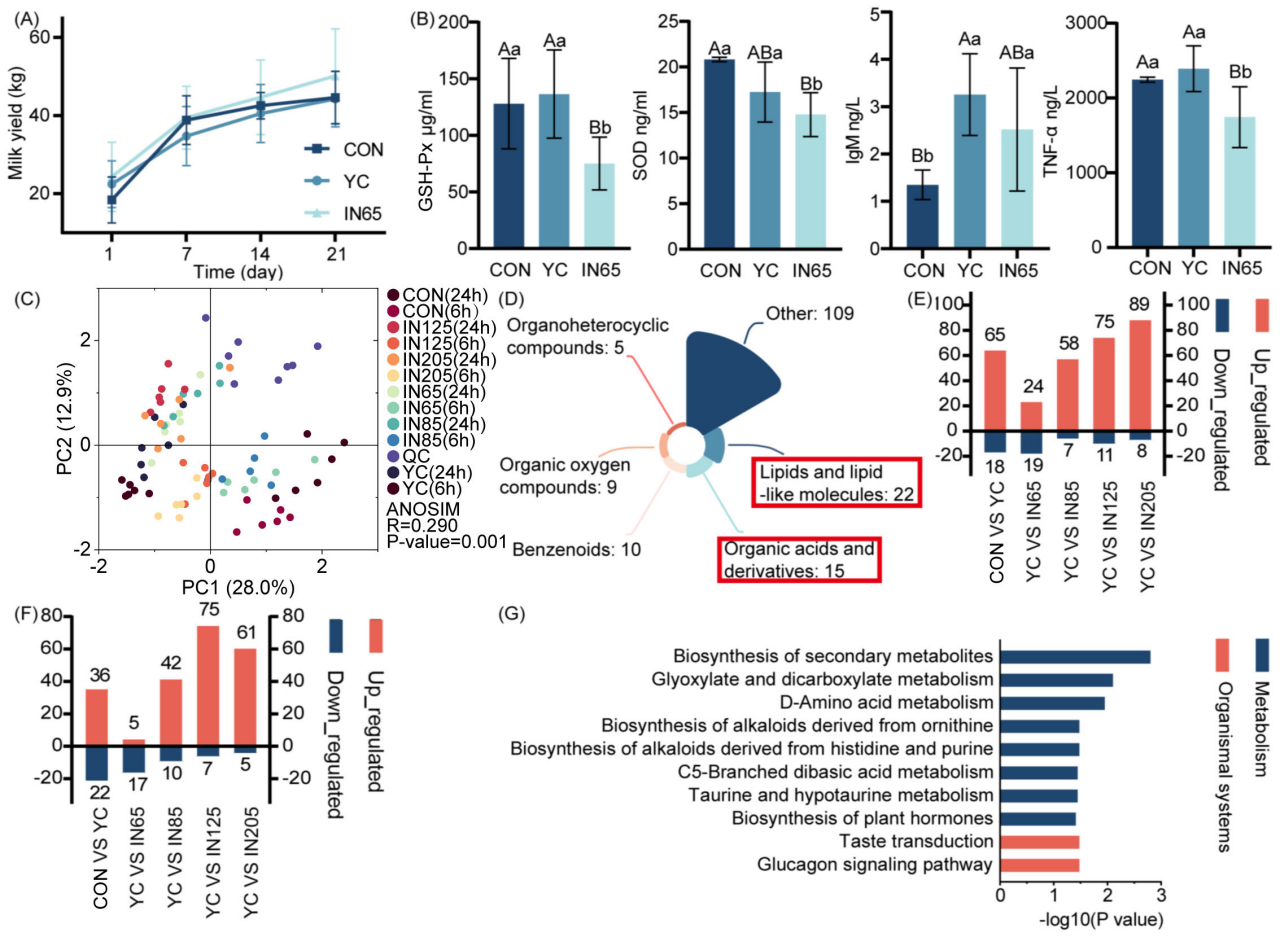
Effects of YC and *myo*-inositol supplementation on milk yields **(A)**, GSH-Px, SOD, TNF-α levels in serum **(B)**, rumen fluid metabolism PCA **(C)**, super class of differential metabolites of the rumen fluid *in vitro* **(D)**, up-regulated and down-regulated metabolites of rumen fluid metabolomics at 6 h **(E)** and 24 h **(F)**, KEGG pathway enriched by differential metabolites in the *myo*-inositol treatment groups compared with YC group *in vitro* **(G)**.

At 21 days postpartum, compared with the control group and YC group, a numerically higher milk yield (+4.00% and +4.05%) was observed in the IN65 group, though statistical significance was not achieved due to limited sample size and high variability (**Figure 2A**). **Table 6** shows no significant differences in oxidation, immune, or inflammatory indicators at calving. On the 21 days postpartum, GSH-Px level in the IN65 group was significantly lower compared to the YC and CON groups (*P* < 0.01), and SOD level was significantly lower than that in the CON group (*P* < 0.01). These findings suggested that myo-inositol affects oxidative stress status in peripartum dairy cows. Following a period of YC or *myo*-inositol feeding, TNF-α levels in the IN65 group were significantly lower than those in the YC group (*P* < 0.01), and IgM levels in the YC group were significantly higher than those in the CON group (*P* < 0.01). From day 1 to day 21 postpartum, SOD, TAS, TOS and IL-10 levels in the CON group showed a significant increase (*P* < 0.01). Notably, this trend was significantly mitigated after the addition of *myo*-inositol (**Table 7**). The findings indicate that *myo*-inositol affected oxidative stress in peripartum dairy cows (**Figure 2B**).

**Table 6:** Oxidation and antioxidant, immune, and inflammation indices in serum of CON cows, YC cows and IN65 cows.

Day	Item	CON	YC	IN65	SEM	*P*-Value
1 d	Oxidation and antioxidant indices					
	CAT ng/ml	19.86	18.58	19.52	0.72	0.78
	MDA nmol/ml	7.66	5.37	6.01	0.43	0.20
	GSH-Px µg/ml	107.13	101.45	93.23	5.94	0.13
	SOD ng/ml	14.47	16.63	14.94	0.87	0.48
	TAS µmol/L	7.74	15.57	16.19	1.94	0.30
	TOS µmol/L	184.86	317.34	213.38	38.37	0.36
	Immune indices					
	IgA mg/ml	0.96	1.33	1.13	0.07	0.12
	IgM mg/ml	3.17	2.80	3.45	0.28	0.55
	Inflammation indices					
	IL-6 ng/L	36.56	45.14	43.99	6.56	0.93
	IL-10 ng/L	3099.61	5016.9	6054.15	472.81	0.09
	IL-Iβ ng/L	189.67	168.31	171.25	11.02	0.84
	IFN-γ ng/L	1918.03	1848.95	1733.38	104.53	0.81
	TNF-α ng/L	1667.93	1934.98	1847.46	88.77	0.77
21 d	Oxidation and antioxidant indices					
	CAT ng/ml	17.59	20.09	16.00	0.86	0.09
	MDA nmol/ml	5.23	5.56	5.24	0.28	0.99
	GSH-Px µg/ml	127.97^Aa^	136.46^Aa^	75.15^Bb^	8.82	<0.01
	SOD ng/ml	20.81^Aa^	17.24^ABb^	14.78^Bb^	0.66	<0.01
	TAS µmol/L	45.39^Aa^	13.10^Bb^	15.72^Bb^	2.87	<0.01
	TOS µmol/L	549.81^Aa^	277.83^Bb^	229.75^Bb^	33.91	<0.01
	Immune indices					
	IgA mg/ml	0.96	1.36	1.08	0.08	0.15
	IgM mg/ml	1.35^Bb^	3.26^Aa^	2.52^ABa^	0.23	<0.01
	Inflammation indices					
	IL-6 ng/L	50.28	91.93	45.14	19.37	0.53
	IL-10 ng/L	6786.15	5877.25	5763.11	541.88	0.80
	IL-Iβ ng/L	162.06	139.67	146.92	5.11	0.33
	IFN-γ ng/L	1656.06	1477.85	1540.29	108.03	0.86
	TNF-α ng/L	2245.50^Aa^	2391.98^Aa^	1745.24^Bb^	87.15	<0.01

**Table 7:** Oxidation and antioxidant, immune, and inflammation indices in serum of CON cows, YC cows and IN65 cows from D 1 and D 21 postpartum.

Item	Group	1d	21d	SEM	*P*-Value
Oxidation and antioxidant indices					
CAT ng/ml	CON	19.86	17.59	0.96	0.11
	YC	18.58	20.09	0.85	0.39
	IN65	19.52	16.00	1.00	0.08
MDA nmol/ml	CON	7.66	5.23	1.25	0.15
	YC	5.37	5.56	0.25	0.50
	IN65	6.01	5.24	0.49	0.45
GSH-Px µg/ml	CON	107.13	127.97	21.85	0.51
	YC	101.45	136.46	7.49	0.02
	IN65	93.23	75.15	6.38	0.10
SOD ng/ml	CON	14.47	20.81	0.38	<0.01
	YC	16.63	17.24	1.08	0.91
	IN65	14.94	14.78	0.44	0.86
TAS µmol/L	CON	7.74	45.39	1.97	<0.01
	YC	15.57	13.10	1.44	0.20
	IN65	16.19	15.72	2.39	0.93
TOS µmol/L	CON	184.86	549.81	44.85	<0.01
	YC	317.34	277.83	40.42	0.64
	IN65	213.38	229.75	33.60	0.71
Immune indices					
IgA mg/ml	CON	0.96	0.96	0.16	0.99
	YC	1.33	1.36	0.07	0.88
	IN65	1.13	1.08	0.09	0.36
IgM mg/ml	CON	3.17	1.35	0.36	<0.01
	YC	2.80	3.26	0.22	0.17
	IN65	3.45	2.52	0.34	0.18
Inflammation indices					
IL-6 ng/L	CON	36.56	50.28	8.90	0.63
	YC	45.14	91.93	19.35	0.26
	IN65	43.99	45.14	14.74	0.24
IL-10 ng/L	CON	3099.61	6786.15	357.28	<0.01
	YC	5016.9	5877.25	377.85	0.34
	IN65	6054.15	5763.11	729.40	0.85
IL-Iβ ng/L	CON	189.67	162.06	18.80	0.19
	YC	168.31	139.67	10.85	0.16
	IN65	171.25	146.92	9.97	0.24
IFN-γ ng/L	CON	1918.03	1656.06	581.38	0.70
	YC	1848.95	1477.85	111.62	0.15
	IN65	1733.38	1540.29	92.76	0.31
TNF-α ng/L	CON	1667.93	2245.5	210.04	0.07
	YC	1934.98	2391.98	77.38	<0.01
	IN65	1847.46	1745.24	114.31	0.67

### 3.4 Analysis of rumen fluid metabolite and pathway *in vitro*

Principal component analysis (PCA) revealed a clear separation of metabolites at 6 h and 24 h (**Figure 2C**). A total of 170 differential metabolites were identified between the 6 h and 24 h groups compared to the CON group, with variable importance in projection (VIP) > 1 and *P* < 0.05. These metabolites were lipids and lipid-like molecules and organic acids and derivatives (**Figure 2D**). At 6 h of *in vitro* fermentation, 65 metabolites were upregulated in the YC group compared to the CON group (**Figure 2E**). At 24 h of *in vitro* fermentation, 36 differential metabolites were found to be upregulated (**Figure 2F**).

The study highlights the need for a comprehensive evaluation of the impact of YC and *myo*-inositol on ruminal fermentation in dairy cows. Further analysis of rumen metabolites and their pathways revealed key metabolic pathways with significant impact. Enrichment analysis identified pathways with *P* < 0.05 and impact > 0.2 as crucial in understanding the effects of these supplements.

The effects of supplementing *myo*-inositol on *in vitro* rumen fermentation in dairy cows were shown by examining the differential metabolites and enriched metabolic pathways between the YC and CON groups. After 6 h of *in vitro* fermentation, analysis of Kyoto Encyclopedia of Genes and Genomes (KEGG) pathways showed that YC mainly affects the biosynthesis of plant secondary metabolites and unsaturated fatty acids. During 24 h of *in vitro* fermentation, KEGG pathway enrichment analysis demonstrated that YC primarily affected the biosynthesis of plant secondary metabolites, terpenoids, and steroids.

Analysis of the differential metabolites and enriched differential metabolic pathways between the YC group and the *myo-*inositol-supplemented (IN65, IN85, IN125, and IN205) groups indicated that the inclusion of *myo*-inositol in the diet affected rumen fermentation in dairy cows. KEGG pathway enrichment analysis after 6 h of *in vitro* fermentation revealed that *myo*-inositol mainly affected AA metabolism, particularly D-amino acid metabolism, and taurine and hypotaurine metabolism (**Figure 2G**). After 24 h of *in vitro* fermentation, KEGG pathway enrichment analysis indicated that *myo*-inositol predominantly affected styrene degradation and fatty acid biosynthesis.

### 3.5 Differential serum metabolite and pathway analysis

**Figure 3:**
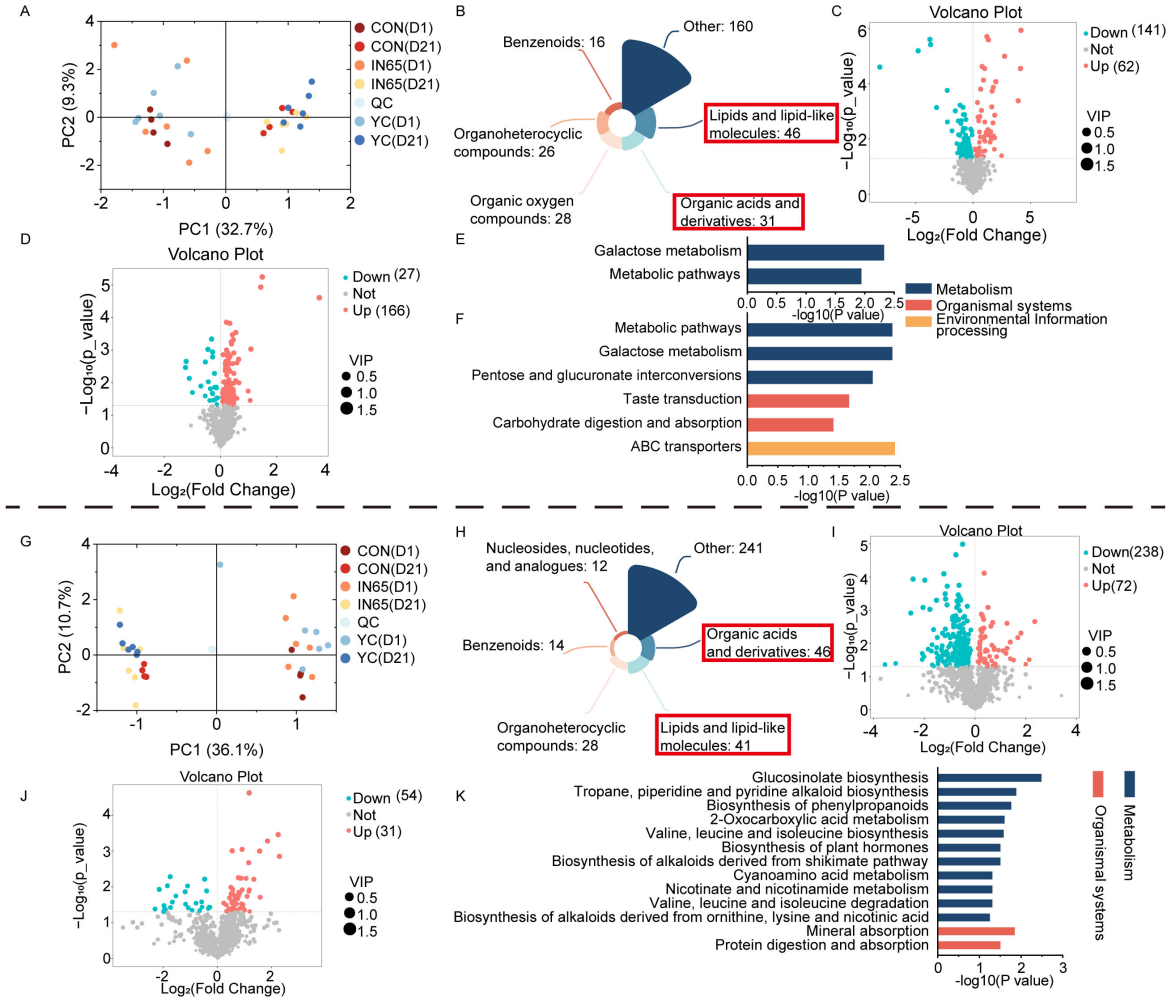
Serum metabolism PCA **(A)**, super class of differential serum metabolites **(B)**, volcano maps of serum metabolites in the YC vs CON **(C)** and IN65 vs YC **(D)**, significantly different KEGG pathways of serum in the YC vs CON **(E)** and IN65 vs CON **(F)**, milk metabolism PCA **(G)**, super class of differential milk metabolites **(H)**, volcano maps of milk metabolites in the YC vs CON **(I)** and IN65 vs YC **(J)**, significantly different KEGG pathways of milk in the IN65 vs CON **(K)**.

PCA analysis showed a clear separation between metabolites from cows at day 1 and day 21 postpartum (**Figure 3A**). In the serum samples of the three groups of dairy cows 21 days after calving, 307 differential metabolites with VIP > 1 and *P* < 0.05 were identified and then classified. The most abundant differential metabolites included lipids and lipid-like molecules, and organic acids and their derivatives (**Figure 3B**). In comparison to the CON group, the YC group had 62 upregulated and 141 downregulated differential metabolites (**Figure 3C**). Metabolic pathways with *P* < 0.05 and impact > 0.2, such as galactose metabolism and other differential metabolic pathways, were identified through enrichment analysis of the pathways where the differential metabolites were located (**Figure 3E**).

In the IN65 group, 166 differential metabolites were upregulated and 27 were downregulated compared to the YC group (**Figure 3D**). Enrichment analysis of the differential metabolites between IN65 and CON showed significant metabolic pathways such as galactose metabolism, pentose and glucuronate interconversions, taste transduction, and carbohydrate digestion and absorption (**Figure 3F**).

### 3.6 Differential milk metabolite and pathway analysis

PCA analysis showed a clear separation between metabolites from cows at day 1 and day 21 postpartum (**Figure 3G**). In the milk samples collected from the three groups of cows 21 days after calving, a total of 382 differential metabolites with VIP > 1 and *P* < 0.05 were screened and then classified. The most differentially metabolites included organic acids and derivatives, lipids and lipid-like molecules (**Figure 3H**). In comparison to the CON group, the YC group had 72 upregulated and 238 downregulated differential metabolites (**Figure 3I**). In comparison to the YC group, the IN65 group had 54 upregulated and 31 downregulated differential metabolites (**Figure 3J**). Enrichment analysis of differential metabolites between the CON and YC groups revealed significant metabolic pathways with *P* < 0.05 and impact > 0.2, such as glucosinolate biosynthesis and tropane, piperidine, and pyridine alkaloid biosynthesis (**Figure 3K**). Additionally, metabolic pathways with *P* < 0.05 and impact > 0.2 were selected through enrichment analysis, including the sphingolipid signaling pathway.

**Figure 4:**
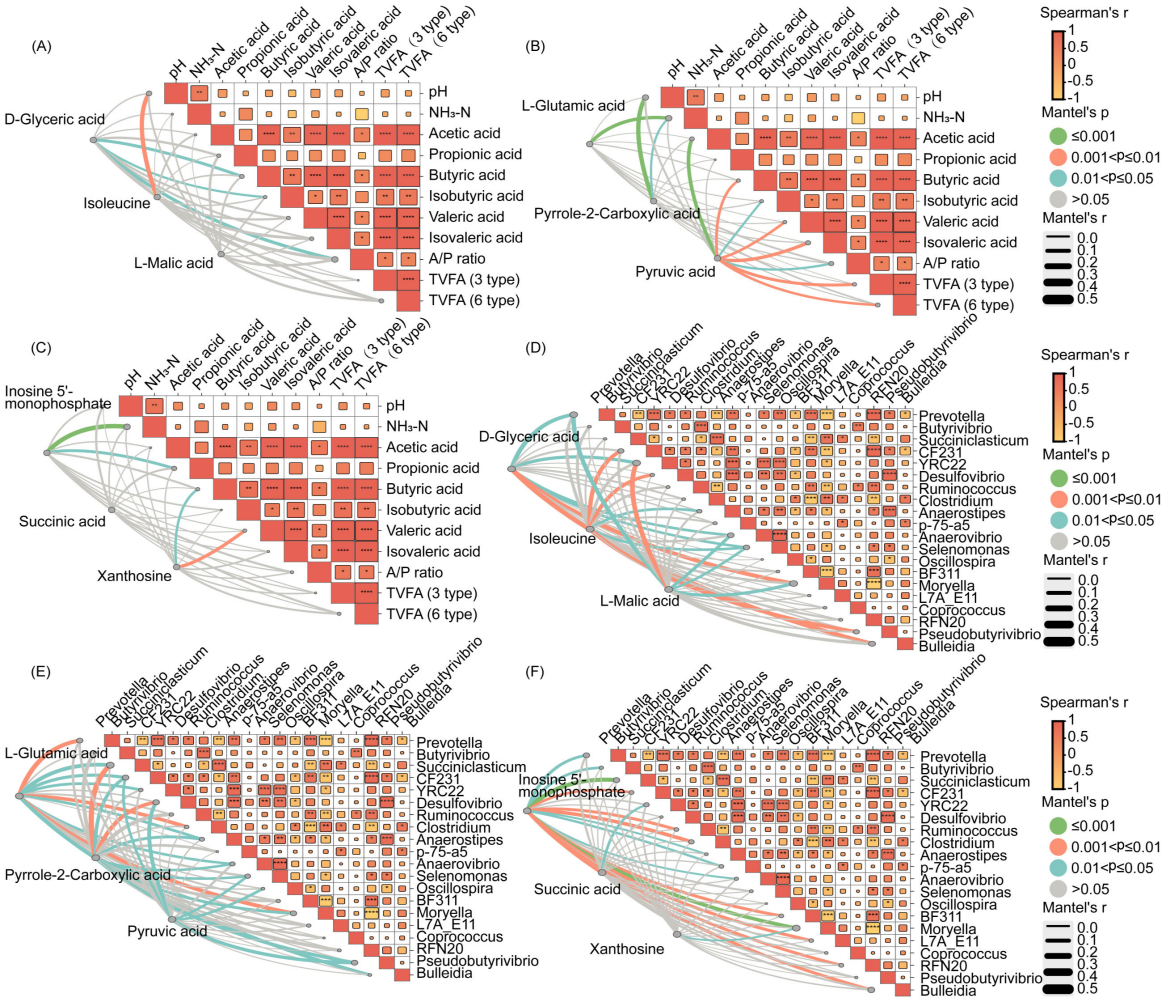
Correlation between fermentation index was analyzed by the Spearman correlation analyses, correlation between fermentation index indicators and nine differential metabolites was analyzed by the Mantel test **(A–C)**. Correlation between microbial genera was analyzed by the Spearman correlation analyses, correlation between nine differential metabolites indicators and microbial genera was analyzed by the Mantel test **(D–F)**.

Correlation analyses were performed between the differential metabolites in rumen fluid, fermentation indicators, and rumen bacteria (**Figure 4**). Acetic acid was positively correlated with D-glyceric acid and xanthosine (**Figures 4A, C**). Similarly, D-glyceric acid, inosine 5-monophosphate, and xanthosine were positively correlated with genus, such as *Bulleidia* and *Moryella* (**Figures 4D, F**). This suggested that myo-inositol affects the production of acetic acid and butyric acid, by regulating the composition of rumen bacteria and their metabolism.

## 4 Discussion

YC is widely used to regulate the ruminal environment in dairy cows. However, as a functional substance in YC, *myo*-inositol is primarily used in aquaculture and is rarely in ruminants. Our research findings indicated that the *myo*-inositol had distinct impacts on rumen fermentation (*in vitro*), health, and milk production compared to the CON group.

Research has indicated that supplementing dairy cow diets with nitrogen sources leads to an increase in the proportion of fiber-degrading bacteria in the rumen (21). In this study, the NH_3_-N levels at 12 h were lower than that at 6 h, possibly because of the increased utilization of NH_3_-N in the rumen by fiber-degrading bacteria.

In this study, the pH of the YC, IN65, IN85, and IN205 groups were lower than that of the CON group after 3 h of *in vitro* fermentation. However, after 24 h of fermentation, the pH of these groups were higher than that of the CON. These findings indicate that *myo*-inositol, helps stabilize rumen pH. Numerous studies have shown that incorporating yeast supplements into the diet can increase the levels of TVFA in the rumen of dairy cows (22). *Myo*-inositol, an effective substance in YC, may have diverse effects. Research has indicated that *Anaerostipes* can metabolize dietary *myo*-inositol to produce propionic and acetic acids (23). In the present study, it was found that IN65 and IN85 had a significant effect on the production of NH_3_-N, acetic acid, butyric acid, isobutyric acid, valeric acid, and isovaleric acid. These effects were most pronounced at 6 h and 12 h. Based on these findings, we conclude that *myo*-inositol affects rumen flora, resulting in changes in the concentrations of several VFA. Genus-level investigations showed that *Moryella*, *Succiniclasticum*, *Clostridiaceae_Clostridium*, and *Bulleidia* had higher relative abundances in the IN65 group compared to the YC group. Among these, *Moryella* can use carbohydrates and proteins to produce lactic, butyric, and short-chain fatty acids (24). *Succiniclasticum*, *Clostridiaceae_Clostridium*, and *Bulleidia* belong to the Firmicutes. They share common functions and can convert cellulose into VFA, thereby promoting digestion, growth, and development (25).

Changes in the metabolism of different AA have been observed in the rumen fluid metabolic pathways. After the addition of *myo*-inositol, a variety of metabolic pathways were changed, affecting differential metabolites like glutamate, ornithine, and histidine, which were found to be enriched in the KEGG differential metabolic pathways. Glutamate are essential for nitrogen metabolism and the TCA cycle in the rumen of ruminants (26). Glutamate, a recognized natural antioxidant, is an AA component of glutathione, another well-known natural antioxidant (27, 28). Ornithine is involved in the synthesis of polyamines in the rumen, which play a crucial role in the growth and activity of microorganisms (29). Histidine residues in proteins can act as buffers to help maintain the pH balance in the rumen. Intravenous injection of different amounts of histidine into lactating cows can lead to changes in feed intake and milk production (30). Mantel analysis was conducted on differentially expressed metabolites, fermentation indices, and microbial abundance to assess their correlation. The result showed a strong correlation between differential metabolites, fermentation indices, and microbial abundance. Therefore, these AA are closely associated with ruminal metabolism in dairy cows.

This study identified several important differential metabolic pathways by enriching differential metabolites from the IN65 vs. YC group. One of these pathways is pentose and glucuronate interconversion, which involves the transport and metabolism of key intermediates in the pentose phosphate pathway and the glucuronide pathway. These processes are essential for supporting cellular functions, detoxification, and redox balance. Research has shown that in mice with a complete knockout of the glutamate-cysteine ligase modifying subunit gene, exposure to the liver results in increased glutamate production, activation of the glucuronide pathway, and stimulation of nucleotide biosynthesis. The increased glutamate production suggests enhanced GSH-Px biosynthesis. The induction of nucleotide biosynthesis can provide nutrients for hexosamine biosynthesis and the glucuronide pathway (31). This pathway is essential for the production of NADPH_2_ and ribose-5-phosphate, which are essential for antioxidant defense and nucleotide synthesis, respectively. The glucuronide pathway involves the conversion of glucose derivatives to glucuronic acid, which plays a key role in the detoxification and conjugation of various substances (32). The study found that the GSH-Px index of the IN65 group was significantly lower compared to the YC and CON groups, possibly due to the activity of pentose and glucuronate interconversions in the blood. *Myo*-inositol can affect the transport of proteins. ABC transporters participate in the transport of nutrients, antioxidants, and metabolites into blood (33). Research on Holstein cows has shown that the proportion of ABC transporters also increases significantly as cows move from the dry period to the lactation period, peripartumly accounting for the increase in milk production observed in the IN65 group 21 days after delivery in this study (15). To explore the specific metabolic changes in milk, metabolomic analysis was performed on milk samples from the IN65, YC, and CON groups.

Analysis of milk metabolites in the IN65 and YC groups demonstrated that inositol influences the sphingolipid signaling pathway in milk. In this study, the main differential metabolites enriched in the sphingolipid signaling pathway in the IN65 group were C14 dihydroceramide, C16 ceramide, and C18 dihydroceramide. Interestingly, ceramide formation can be induced by different stimuli, such as TNF-α (34, 35). Ceramide can also be produced by sphingomyelin hydrolysis, a process affected by TNF-α or oxidative stress (36). This suggests that the reduction in TNF-α in the IN65 group was achieved by affecting the sphingolipid signaling pathway.

This study confirmed the effects of *myo*-inositol on rumen fermentation indicators, microbial flora, and metabolism through *in vitro* research. Additionally, it also determined the most effective dosage of *myo*-inositol supplementation through *in vitro* experiments. This optimal dosage was subsequently applied in animal experiments, and a metabolomic analysis of blood and milk was conducted to evaluate its effects on oxidative stress, immune response, and inflammatory indicators.

## 5 Conclusion

The IN65 was an optimal dose *in vitro*, which led to an increase in the concentration of VFA and the relative abundance of ruminal *Bacteroidetes*. Furthermore, *myo*-inositol was observed to primarily affect amino acid metabolism in the rumen, glucose metabolism in the blood, and sphingolipid metabolism in milk. These findings provide a theoretical basis for the use of *myo*-inositol as an additive to increase milk production in peripartum dairy cows.

## Data Availability

In this study, all the data generated or analysed are included in this paper. The 16s rRNA data presented in the study are deposited in the NCBl Sequence Read Archive repository, accession number PRJNA1101182.
